# Entropy Drives Interpolymer Association in Water: Insights into Molecular Mechanisms

**DOI:** 10.1021/acs.langmuir.3c02978

**Published:** 2024-03-22

**Authors:** Tobias Benselfelt, Goksu Cinar Ciftci, Lars Wågberg, Jakob Wohlert, Mahiar Max Hamedi

**Affiliations:** Department of Fibre and Polymer Technology, School of Engineering Sciences in Chemistry, Biotechnology and Health, KTH Royal Institute of Technology, 100 44 Stockholm, Sweden

## Abstract

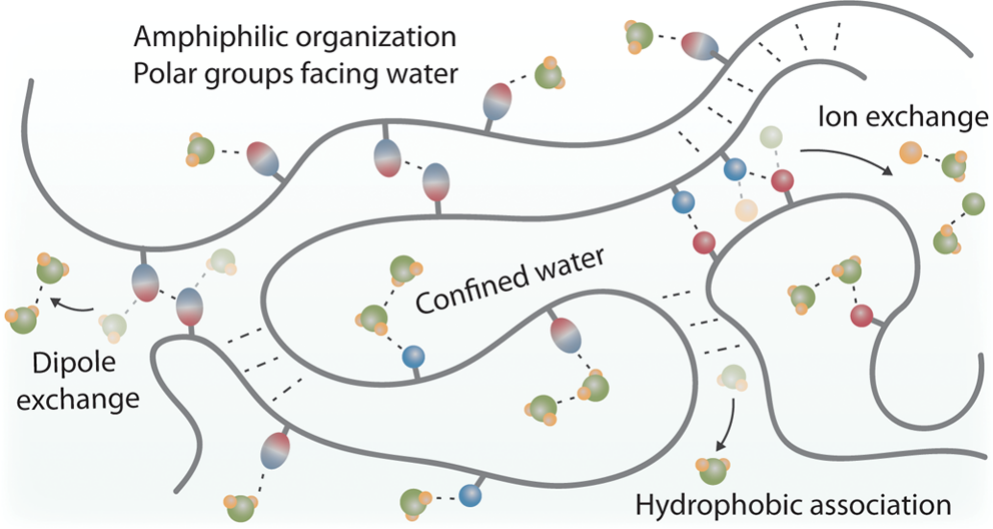

Interpolymer association in aqueous solutions is essential for many industrial processes, new materials design, and the biochemistry of life. However, our understanding of the association mechanism is limited. Classical theories do not provide molecular details, creating a need for detailed mechanistic insights. This work consolidates previous literature with complementary isothermal titration calorimetry (ITC) measurements and molecular dynamics (MD) simulations to investigate molecular mechanisms to provide such insights. The large body of ITC data shows that intermolecular bonds, such as ionic or hydrogen bonds, cannot drive association. Instead, polymer association is entropy-driven due to the reorganization of water and ions. We propose a unifying entropy-driven association mechanism by generalizing previously suggested polyion association principles to include nonionic polymers, here termed polydipoles. In this mechanism, complementary charge densities of the polymers are the common denominators of association, for both polyions and polydipoles. The association of the polymers results mainly from two processes: charge exchange and amphiphilic association. MD simulations indicate that the amphiphilic assembly alone is enough for the initial association. Our proposed mechanism is a step toward a molecular understanding of the formation of complexes between synthetic and biological polymers under ambient or biological conditions.

## Introduction

Interactions between macromolecules result in condensed phases in water, also known as interpolymer complexes.^1,2^ These interactions are essential in the chemistry of life, such as protein folding and molecular recognition, DNA duplexing, or assembly of the plant cell wall components.^3−5^ It has even been proposed that dense liquid phases of polymers, known as coacervates, provided a high enough local concentration of molecules for life to originate in the vastly dilute primordial oceans.^6^ In material and colloidal science, these interactions have been exploited to direct the assembly of polymers, biological and synthetic,^7^ for example, using the layer-by-layer self-assembly technique.^8,9^

Despite the vital importance of interpolymer interactions in water, there is still no consistent and unified description of the driving forces behind their association. Classical theories, such as those by De Gennes, Scheutjens, Fleer, or Flory and Huggins, have successfully described many systems semiquantitatively.^10^ However, these theories are based on mean-field approximations, meaning that molecular details are lost. Important mechanisms are gathered in attraction parameters, such as Tδ or χ, which provide no information about what is happening at a molecular level. Some more recent thermodynamic models require system-specific constants/parameters that hide most molecular details.^11,12^ With emergent molecular dynamics simulations, molecular details are increasingly available to provide a complete picture.

Scientists have discussed molecular details, regardless of the shortcomings of mean-field theories. The current consensus for ideal polyion association is an ion exchange process driven by the entropy gained upon the release/reorganization of counterions and associated water.^13,14^ Similarly, hydrophobic polymers associate due to the entropy or enthalpy change from reorganizing structured water and minimizing the area of exposed surfaces that induce this structuring, commonly called the “hydrophobic effect.” However, the behavior of polymers carrying properties between the two extremes of polyions and hydrophobic polymers, e.g., polar nonionic polymers, here termed polydipoles (any polymer with polar monomers that are not ionized), is more ambiguous. Polydipoles and polyions are thus treated separately in most thermodynamic discussions. Here, we argue that this is unnecessary and that there is a general association mechanism.

The polarity of water-soluble polydipoles often leads to the conclusion that the formation of hydrogen bonds (H-bonds) drives their association.^1,9^ What is neglected in this discussion is that interactions between polydipoles are often endothermic, which disqualifies H-bonding as the driving mechanism, since a bond is enthalpy-driven (see our definition in Table 1) and thus cannot drive an endothermic process. Previous experiments show significant positive changes in entropy upon the association, which has been assigned to “hydrophobic effects”.^1,15^ The literature data thus suggest that polymer association in water is generally not due to specific intermolecular bonds. Instead, it is driven by the entropy of reorganizing ions or solvent molecules. Any entropic changes due to solvent reorganization resulting from the bond are separate mechanisms that need to be understood. Recent MD simulations have shown that the reorganization of water drives the association of charged and uncharged polymers,^15−17^ and this reorganization is also suggested as the main driving force for molecular recognition.^5^

**Table 1:** Definitions of the Associative Mechanism between Polymers

terminology	definition	driving	opposing	comment
bond (electrostatic forces)	electrostatic forces acting between two molecular species lead to aggregate formation	enthalpy	entropy	a bond can only form if the enthalpy change is negative, as it reduces entropy. Bonds are directional and temperature-dependent
ionic bond				
covalent bond		Δ*H* < 0	Δ*S* < 0	
hydrogen bond				
polar bond				
electrodynamic forces	electrodynamic forces acting between two molecular species lead to aggregate formation	enthalpy	entropy	not directional and temperature-independent
dispersion forces				
Debye forces		Δ*H* < 0	Δ*S* < 0	
attractive interaction	description of attraction between molecular species considering all components of the system	enthalpy	entropy	the sum of all thermodynamic changes results in a negative free energy (Δ*G* < 0). Endothermic interactions (Δ*H* > 0) cannot be driven by bonds
solvophobic effect		Δ*H* < 0	Δ*S* < 0	
charge exchange		entropy	enthalpy	
amphiphilic association		Δ*S* > 0	Δ*H* > 0	
nonequilibrium interaction	an interaction trapped in a local Δ*G* minimum due to conformational restrictions			it can take an infinite time for polymers to conform to equilibrium. The state in which the polymers are trapped can depend on concentration or in which order they are introduced

To understand why intermolecular bonds are unlikely to drive association in water, we note that these bonds originate from electrostatics (ionic, polar, H-bonds) and that water, being a highly polar solvent, effectively screens these electrostatic bonds: salts dissolving in water is an example. As a result, interpolymeric polar bonds compete with polar bonding to water, which is favored by the significantly higher conformational and translational freedom of water molecules compared to that of the polymer backbone. For example, water is the best hydrogen-bonding liquid, leading to the inevitable question: why would it be more favorable to form hydrogen bonds with something else? The most reasonable answer is a high entropic penalty for a solvent with high degrees of freedom to form restrictive bonds with or around less mobile macromolecules. If these bonds can be exchanged to something with fewer degeress of freedom, like another polymer, such an exchange can greatly increase the entropy of the system.

Suppose intermolecular H-bonds are strong and favorable in water. If so, all polar molecules would be strongly associated, and the dynamic intermolecular chemistry of life, as we know it, would not exist. Instead, a combination of weak H-bonds in the correct positions with significant hydrophobic effects has been put forward as the most reasonable explanation for biological affinity.^5^ The direct enthalpic contribution of the H-bonds is negligible, and its significance has been debated. Advanced receptor design requires a combination of hydrophobic effects, with precisely positioned H-bonds having an enthalpy lower than that of the binding water, to favor the association. Most polymers are, however, less complicated, and their association requires a more straightforward explanation.

**Figure 1:**
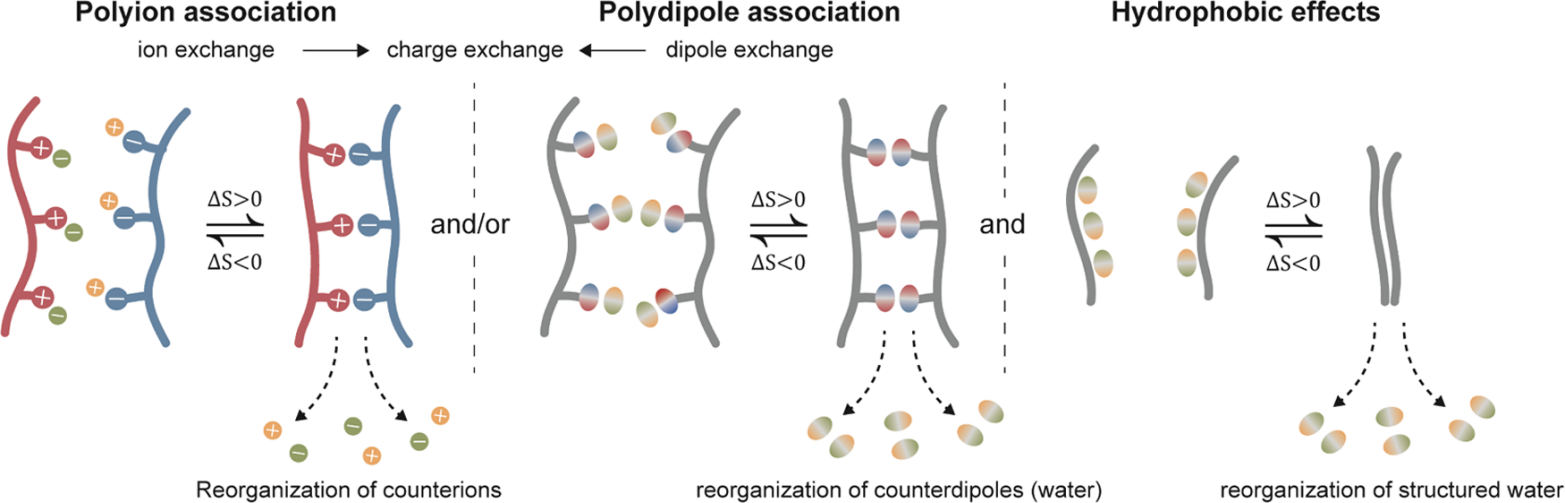
Simplified schematic representation of the mechanism of polyion association in water by the entropic gain of reorganizing counterions (left) from confinement close to the polyion, the similar mechanism we propose for polydipole association by the entropic gain of reorganizing counterdipoles (middle), and purely hydrophobic effects by reorganizing structured water (right). Note that a reorganization can be translational and conformational, and the description presented above is just a simplified schematic cartoon. The central concept is that counterions or solvent molecules escape unfavorable confinement by charge exchange when the polymers associate, which favors entropy.

In this work, we discuss such a mechanism by consolidating previous literature with complementary isothermal titration calorimetry (ITC) measurements where needed and molecular dynamics (MD) simulations to investigate molecular details. The data shows that increased entropy is the main contributor to the favorable free energy of polymer association in water under ambient or biological conditions. Based on this, we propose a charge exchange mechanism for both polyions and polydipoles (Figure 1) unifying polymer association in water. Here, all polar polymers, polyions and polydipoles, are carriers of particular charge densities compensated by counter-charge densities, either dipoles (water) or ions. The exchange of compensating charges leads to an increase in entropy, i.e., entropy-driven charge exchange. Combined with a hydrophobic (amphiphilic) assembly, it leads to a coherent mechanistic model, although challenging to show experimentally due to a lack of resolution in water.^5^

## Results and Discussion

While processing the data, we realized that the nomenclature and definitions are potential sources of confusion in these discussions. To avoid confusion, Table 1 defines the terminology we use here.

Some may object to Table 1 and argue that a bond is described by both an electrostatic force and reorganization of molecules, similar to the chi-parameter in Flory–Huggins solution theories, which contains the bond enthalpy and entropies other than the entropy of mixing, i.e., vibration, rotation, translation, etc. However, defining a hydrogen bond as having both enthalpic and entropic contributions from the solvent does not help our understanding, as we must include the entire system in the bond. Certainly not the intention of the terminology. With such a definition of a bond, the idea that something is driven by bonds becomes entirely irrelevant and may even be misleading. If a bond is defined by a bond enthalpy, i.e., energy that needs to be added to break it, it is strictly enthalpy-driven. For a deeper understanding of association mechanisms, we must discuss enthalpy and entropy separately, which is why we distinguish between a bond and an attractive interaction.

We also note that this work deals with ambient temperature and pressure or those found in biological systems, which are the environments in which we often discuss polymer association. Extreme environments will certainly change things, such as a transition to enthalpy-driven association at high temperatures.^12,15^ However, this will not change the molecular mechanisms, just the energy balance.

### Polyion Association–Ion Exchange

In 1956, Michaels described polyion association as driven by “the escaping tendency of the microions associated with each separate polyion”.^14^ Since then, there have been numerous studies on polyion association in which calorimetric techniques such as ITC allowed the determination of thermodynamic parameters. Although polymer association is a kinetically trapped nonequilibrium interaction, meaning that their coil conformation prevents them from reaching true equilibrium within a reasonable time (see Table 1), one can assume that the difference between the trapped state and equilibrium is reasonably small in dilute solutions of low molecular weight polymers. Thermodynamic parameters thus reveal the interaction mechanism between repeat units. However, the nonequilibrium can, in some cases, significantly impact titrations, and ITC data should always be treated carefully.^18^ Despite this, the sign and order of magnitude of the enthalpies and entropies can indeed describe the interaction mechanism.

**Figure 2:**
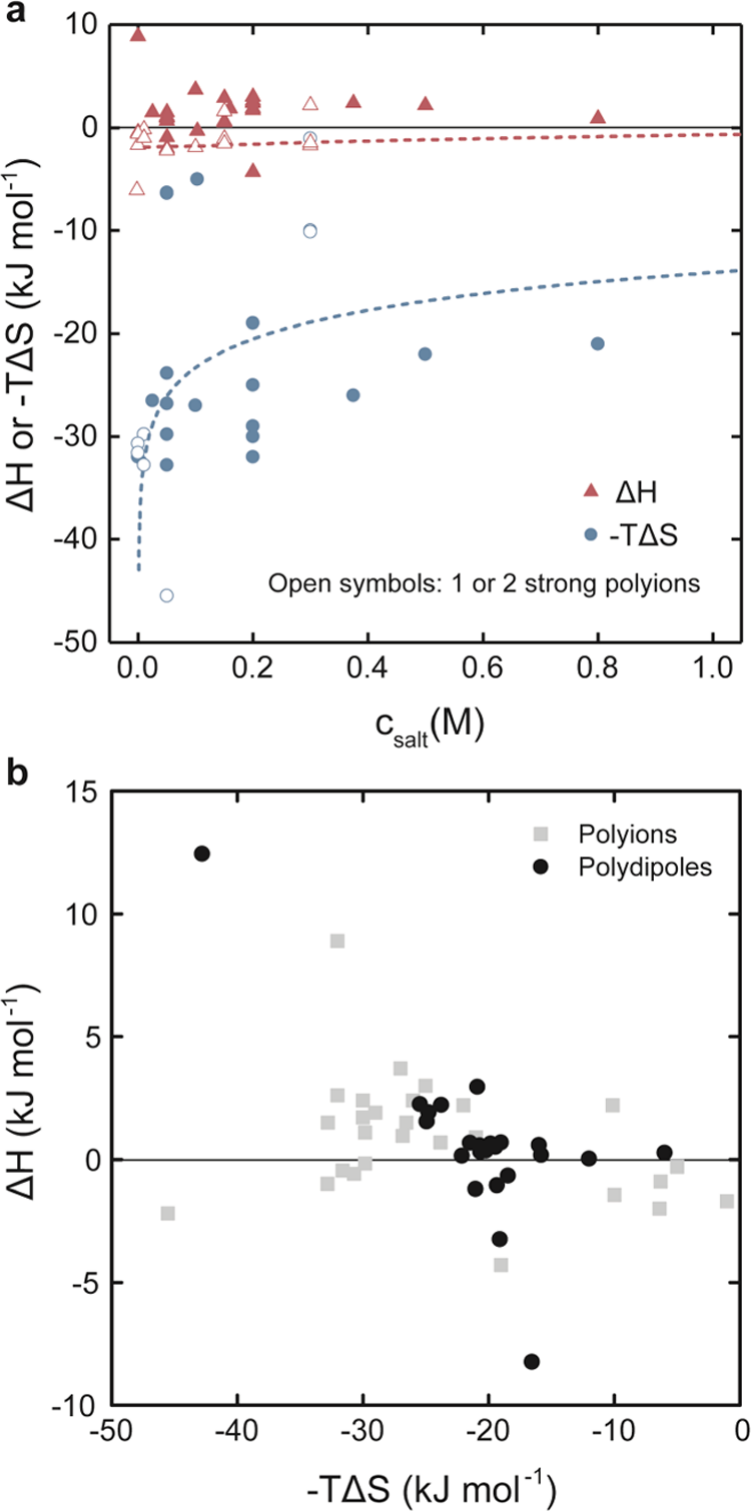
Literature values (references in Table S1 and S2) showing: (a) Enthalpy and entropy contributions for polyion association as a function of background salt concentration. The dashed lines are from a recalculated theoretical model for the association of PDADMA and PSS in NaCl.^13^ Open symbols represent polyion association involving at least one strong polyion, i.e., charged independently of pH. (b) Enthalpy and entropy contributions for polyions (gray squares) and polydipoles (black circles). The relation Δ*G* = −*RT* ln *k* was used to estimate the contribution of entropy when the association constant was given in the literature data.

We have collected thermodynamic literature data for different polyion pairs and some smaller charged molecules in Figure 2a (Table S1), showing Δ*H* and −*T*Δ*S* as functions of the background salt concentration. As a comparison, the thermal energy (*k*_B_*T*) at 25 °C is about 2.5 kJ mol^–1^, and the energy of association (Δ*E*) must be substantially larger than this value for the association to occur according to the probability of states from the Boltzmann distribution (exp(−Δ*E*/*k*_B_*T*)). The data in Figure 2a shows that the entropy term (−*T*Δ*S*) is strictly negative and about an order of magnitude greater than the enthalpy so that more than 90% of the free energy change comes from the increased entropy upon there organization of counterions and associated water.^13^ Conversely, the enthalpy term Δ*H* is close to the thermal energy and can be negative (exothermic) or positive (endothermic).

Previous work by Schlenoff and co-workers^13^ suggests that differences in Δ*H* are due to the change in perturbation of water by the counterions and the charged groups when polyions associate. These differences result in so-called H-bond defects, i.e., small deviations from an ideal H-bond mainly resulting from a small change in bond length or angle. Exothermic association means fewer H-bond defects, whereas endothermic association means more H-bond defects per repeat unit in the associated state. Each defect is minor, but many defects sum up to an effective loss of bond energy equaling a few ideal H-bonds for the system as a whole, even though the number of H-bonds remains constant.

As described by open/closed symbols in Figure 2a and the color mapping in Table S1, the association of nontitrating strong polyions is mostly exothermic. In contrast, the association of weak polyions is mostly endothermic, suggesting that a change in the environment of titrating groups, uncharged polar groups, or counterions of titrating weak polyions increases the number of H-bond defects in the associated state. A sacrifice of H-bonding in favor of gain in entropy can best describe this mechanism.^19^ In other words, enthalpy–entropy compensation when suboptimal H-bonding between polymers compensates for the H-bonds to water, the former restricted by the nonequilibrium state of polymer association. Sacrificing the H-bond energy to favor entropy requires heat, and the association is thus endothermic.

We have indeed previously shown this mechanism for the endothermic association between the two biopolymers, xyloglucan and cellulose, where we noted that the number of hydrogen bonds did not change before and after association.^15,20^ These insights support the view that the state of water is more important than specific electrostatic bonds and that the reorganization of water, the counterdipole, favors polydipole association. The general principle is to move the focus from the polymers toward interfacial water, where the most dramatic change is.

### Polydipole Association–Dipole Exchange

When the interactions between polydipoles are discussed, it is often termed hydrogen-bonded assembly without scientific support for this claim,^9,21^ and when, in fact, experimental evidence suggests the opposite. For example, in the comprehensive overview by Tsuchida and Abe from 1982,^1^ PAA–PEO and similar pairs are placed under the section “complexes formed by hydrogen-bonding.” However, the same section includes data showing that the Δ*H* of the interaction is positive in water, suggestively due to the hydrophobic effects. The presented thermodynamic data were similar to those of weak polyions and instead suggest an increased amount of H-bond defects in the associated state, i.e., the opposite of H-bond-driven association. This example and many others show that hydrogen-bond-driven assembly in water has survived as an explanation for dealing with these polymers. For example, it is frequently suggested that DNA assembles in double helices and that glucan chains assemble into cellulose through hydrogen bonding, but these claims are now being questioned.^4,22^

It is essential to emphasize that hydrogen bonds can form and break. Their energy is, however, generally not in favor of association in water as they merely exchange in the least detrimental way to favor entropy: polydipole-polydipole ⇌ polydipole–counterdipoles ⇌ counterdipole–counterdipole. The number of H-bonds cannot significantly change since it is indeed very costly in terms of energy to leave a possibility for H-bonds unbound in a protic solvent like water. They can, however, have a slight change in bond strength depending on the chemistry of the donor–acceptor pair, marginally contributing to the energy of association.^23^ Also, massively H-bonded networks, for example, formed by drying, tend to stick together once formed, an example of a trapped state.^22^

**Figure 3:**
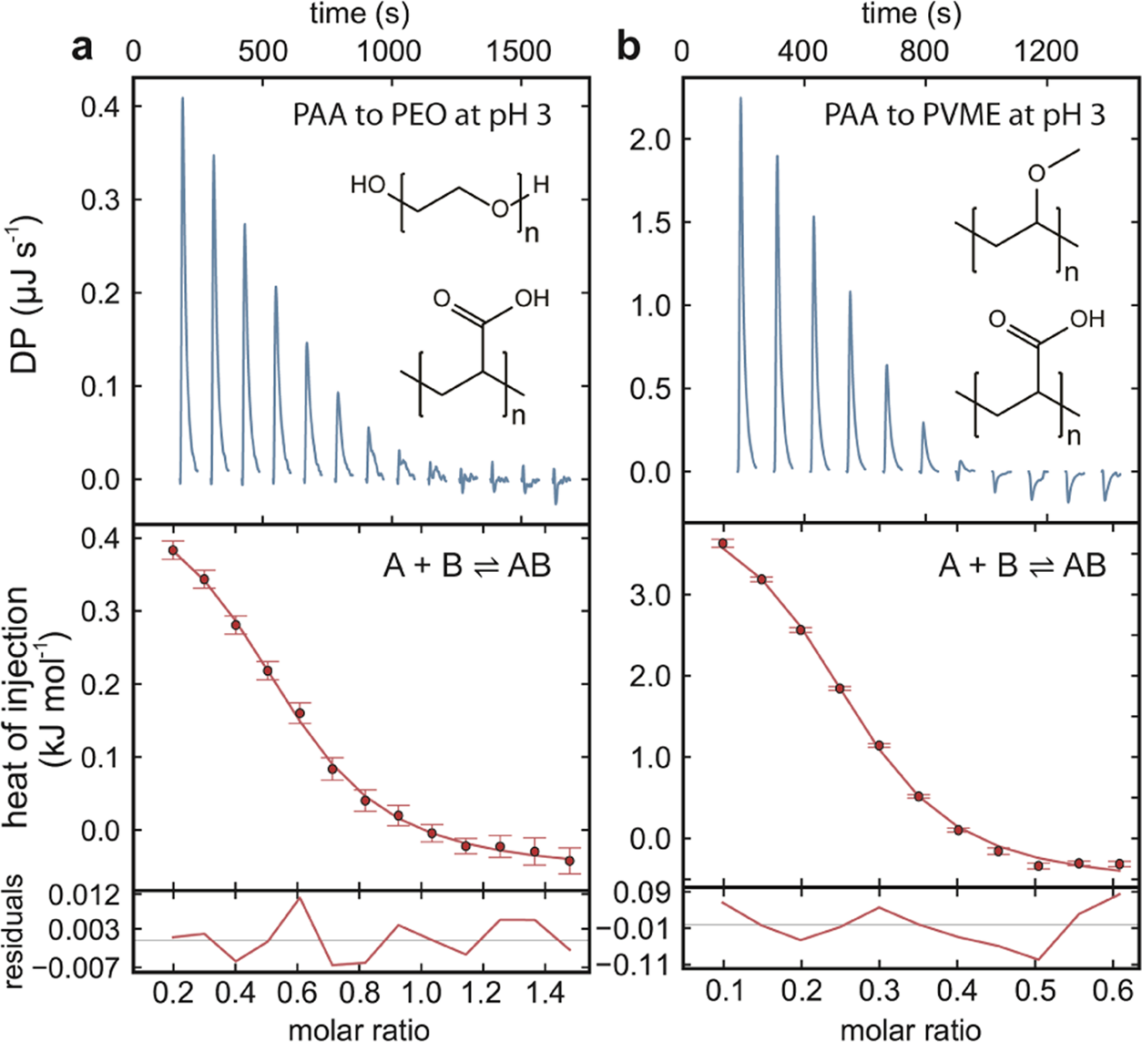
ITC data of (a) the injection of 2 μL of PAA 10 mM repeat units to 203 μL of PEO 1 mM repeat units and (b) the injection of 1 μL of PAA 10 mM repeat units to 203 μL of PVME 1 mM repeat units. Conditions: pH 3 and 25 °C. Data point error bars are errors from the integration, and the solid line is the fitted model with the residuals shown below.

In Figure 2b (listed in Table S2), we gathered thermodynamic data from the literature for different polydipole pairs and compared them to the same data for polyion pairs. We calculated Δ*G* and Δ*S* from the association constant for many of these cases, and to complement the data in the literature with additional fitted values of the entropy, we used ITC to determine thermodynamic parameters by injecting PAA into PEO or poly(vinyl methyl ether) (PVME) at pH 3 (see Figure 3, and Figure S1 for DLS data of the initial solutions). To minimize the influence of the inherent nonequilibrium of the interaction, we used polymers with moderate molecular weights aiming for 100 000 g/mol in dilute solutions with concentrations of 1 or 10 mM of repeat units, corresponding to a concentration range of 0.1–1 g/L.

Figure 2b and typical ITC curves in Figure 3a,b show that polydipole association is mainly endothermic, meaning that H-bonding does not drive the association in an enthalpic sense. Even in exothermic cases, the change in enthalpy is on the order of the thermal energy (*k*_B_*T*), which is not enough to cause association. Instead, entropy is the main contributor to the change in free energy, a general behavior for the interactions between many nonionic substances in water.^22,24,25^

A heteroassociation model (A + B ⇌ AB) was enough to explain the ITC data, and thermodynamic parameters from this model are listed in Table 2, showing that a large entropic change of −*T*Δ*S* ∼−25 to −30 kJ mol^–1^ explains the favorable free energy of association of Δ*G* ∼−25 kJ mol^–1^. Notably, the thermodynamic parameters for polydipole association are in the same order as those reported for polyion association (Figure 2b and Tables S1 and S2), indicating similarities in the mechanisms. Figure 3a,b also shows that PAA and PEO associate in close to a 1:1 molar ratio of repeat units. In contrast, for PAA and PVME, this ratio is closer to 0.5 with an order of magnitude greater Δ*H* (more endothermic), showing that the ratio of complementary polar groups able to form electrostatic bonds between the polymers is not necessarily important, contrary to what would be expected if H-bonds were the driving force. The fact that PVME is more hydrophobic and more favorably associated with PAA suggests that amphiphilic assembly is more important than expected.

**Table 2:** Thermodynamic Parameters for Polydipole Association at pH 3 and 25 °C^a^

polymer pair		*K*_a_ (M^–1^)	Δ*H* (kJ mol^–1^)	–*T*Δ*S* (kJ mol^–1^)	Δ*G* (kJ mol^–1^)
PAA to PEO	mean (*n* = 3)	1.3 × 10^4^	+0.6	**–**24	–24
	std dev	0.1 × 10^4^	0.0	0.2	0.2
PAA to PVME	mean (*n* = 3)	3.4 × 10^4^	+5.1	**–**31	–26
	std dev	0.8 × 10^4^	0.5	0.0	0.5

a The mean and standard deviations are based on three separately fitted titrations.

Let us continue by asking: should polyions and polydipoles be treated separately when they are all polar polymers and show similar association behavior? Polarity is a continuous scale in which 1 e is one ionic charge, but does this definition of charge matter for interaction mechanisms? For example, the dipole moment of an isolated O–H bond is 1.5 D, and two opposite elementary charges at the same separation have a dipole moment of 4.6 D, which, in simplified terms, means that the oxygen and hydrogen in a hydroxyl group have a partial charge equivalent to 30% of the elementary ionic charge (0.3 e). A more striking example is the oxygen of poly(ethylene oxide) oligomers (PEO) or dimethyl ether that can carry a partial charge of up to 0.5 e,^26^ i.e., a half-ion. PEO is indeed one of the most frequently studied polymers involved in polydipole association.^1,9^

Even for the 1 e ionic charge, the situation is not straightforward due to the polarizability of ions and deviations from the assumption of point charges.^27^ A better description of polarity should be charge density in which a dipole has a lower charge density than an ion. As an example, even ions are different. Li^+^ has about eight times higher charge density than K^+^, and indeed, they behave differently, even though they are both monovalent ions according to the definition of ionic charge in mean-field theories.

By referring to both polyions and polydipoles as polymers with a particular charge density, it would make sense that their interaction mechanisms follow similar thermodynamic principles. These principles are a charge exchange in combination with an amphiphilic association in which the increased freedom of counterions and counterdipoles (solvent) drives the association.

Further detailed discussions are found in the Supporting Information, where we discuss ITC data of weak polyion pairs and polyion–polydipole interactions, i.e., a combined ion and dipole exchange. In the Supporting Information, we also discuss extending the charge exchange principles to solvents other than water based on existing data.

### Charge Exchange or Amphiphilic Association; What Is More Important?

As previously mentioned, hydrophobic effects have been suggested to contribute to polydipole association. It is, however, erroneous to call it hydrophobic since the interaction with water is relatively favorable for many polydipoles. It should instead be characterized as an amphiphilic association. After all, polydipoles are soluble in water, and strong self-association is a typical hydrophobic effect. In contrast to purely hydrophobic effects, amphiphilic association leads to a directional assembly due to a favorable orientation of hydrophilic groups toward bulk water.

**Figure 4:**
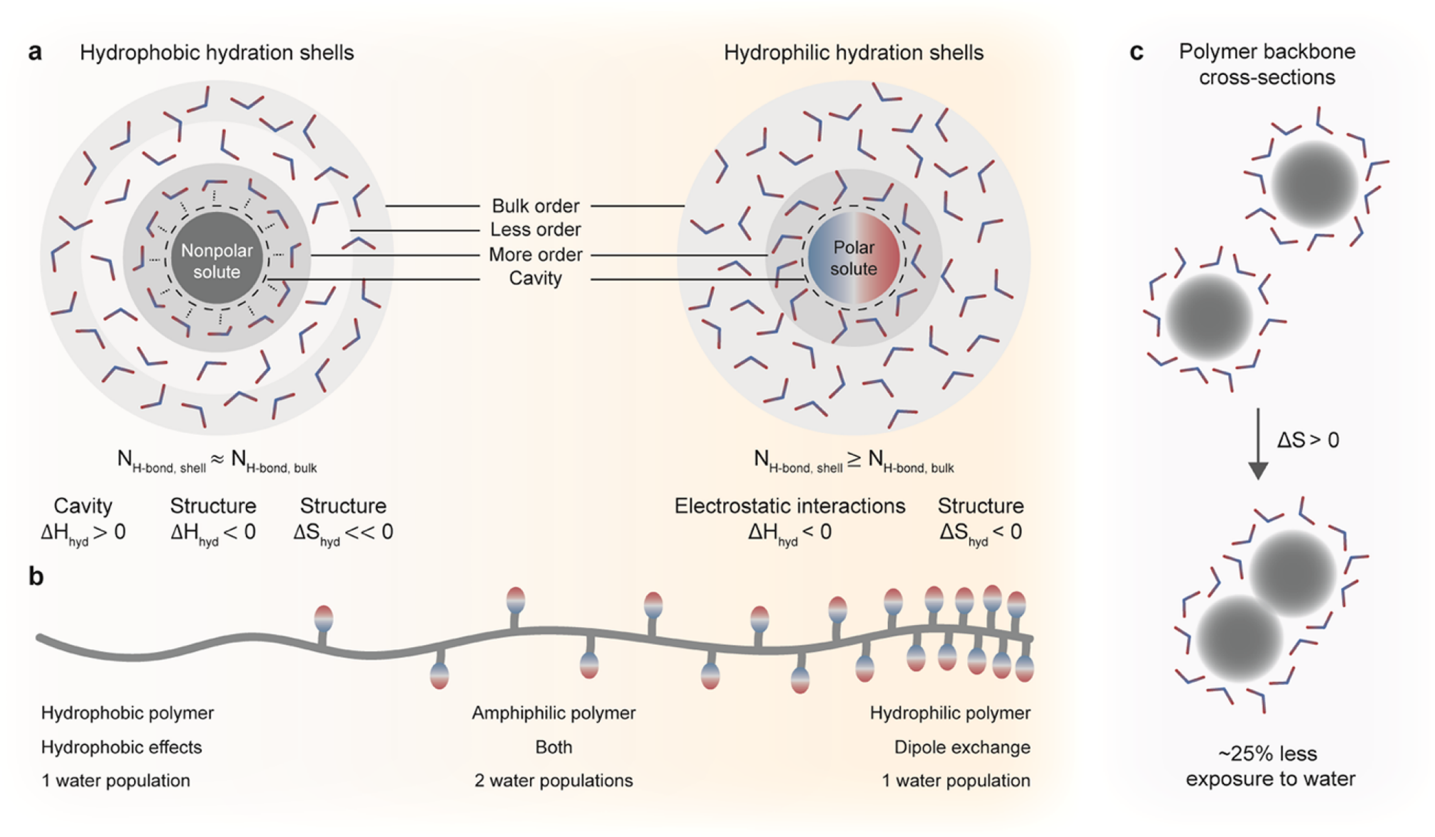
(a) Simplified hydration shells of nonpolar and polar solutes. The hydration shells make the dissolution favorable due to enthalpy at the cost of a structure or confinement of water molecules, which is unfavorable due to entropy. The extreme case of hydrophobic hydration would be that all water molecules are parallel to the solute to form a cage with four H-bonds per water molecule, leaving no H-bonding with the next layer of water molecules.^29^ The depicted hydrophilic hydration layer indicates how the asymmetry of water results in differently hydrated anionic and cationic poles.^32^ Hydrophobic and hydrophilic hydration shells are linked to the terminology of chaotropic (breaker) and kosmotropic (maker) solutes, with respect to the H-bonded network of bulk water. (b) Polymers have properties in the spectrum between hydrophobic and hydrophilic, and hence, at least two different water populations coexist for most water-soluble polymers. (c) Regardless of the interaction mechanism, limiting the exposure to water favors association by increasing the entropy of water.

The difference between hydrophobic/amphiphilic effects and dipole exchange cannot be observed in the thermodynamic data. It can, however, be based on our understanding of how the molecules interact with water (Figure 4a). Hydrophobic effects can be explained by a cavity that disrupts the water network and structured hydration layers with a sustained or improved H-bonded network.^28,29^ The state of the hydrogen-bonded network, however, depends on the size of the hydrophobic solute. The hydrophobic effect can become enthalpy-dominated for particles larger than roughly 1 nm due to the broken H-bonds in the water network.^30^ We argue that polymers fall into the small molecules category since their chain restricts their collapse into a solid particle, and the defining unit is the typically small monomers. Thus, for hydrophobic polymers, the hydration shell favors dissolution by a negative enthalpy of hydration (Δ*H*_hyd_), meaning that the association by the hydrophobic effect is endothermic at room temperature. Here, the unfavorable entropy of the hydration shell (Δ*S*_hyd_) drives the association. Another explanation is that when a hydrophobic complex grows, the characteristic size becomes larger than 1 nm with an enthalpically unfavorable interface to water. As a polymer with a characteristic size of less than 1 nm is added to the complex, entropy is gained and enthalpy is lost because of the increased enthalpically unfavorable surface area.

In contrast, the water molecules surrounding a polar solute are favorably associated by electrostatic bonds, such as H-bonds, and these water molecules can be exchanged to another polar group if it favors entropy (charge exchange).^15^ In both cases, entropy drives the association, and thermodynamic quantities can be similar even though the molecular mechanisms are opposite in the behavior of water, i.e., hydrophobic or hydrophilic. It is, however, probable that during the association of amphiphilic polymers, ion or dipole exchange and amphiphilic effects (Figure 1) coincide since there are different populations of water along a polymer backbone (Figure 4b). Regardless of the energy of these populations, they are often unfavorable states for water. In a simplified scenario (Figure 4c), association reduces the area exposed to water by around 25%, freeing water molecules, which increases entropy.^31^

### MD Simulations–Dipole Exchange or Amphiphilic Effects?

ITC can only reveal the overall thermodynamic change from which we can derive some information, for example, that electrostatic bonds are not of significant importance in an endothermic process.^13^ ITC cannot, however, reveal the molecular mechanism behind this change. Polyion, polydipole, and amphiphilic associations have similar titration data. Classical mean-field theories or recent analytical models are not of much help since all unknown molecular details are clamped into interaction parameters. To find more clarity, we thus turn to molecular dynamics simulations.

Unfortunately, modeling these systems is associated with severe difficulties, as simulating realistically large polymers with atomistic detail is still too demanding regarding computer power. Therefore, we are in a difficult situation with limited experimental and theoretical resolution to study these effects and insufficient computational power to simulate complete systems. We thus need simplifications that still represent the essential features of the molecular system.

**Figure 5:**
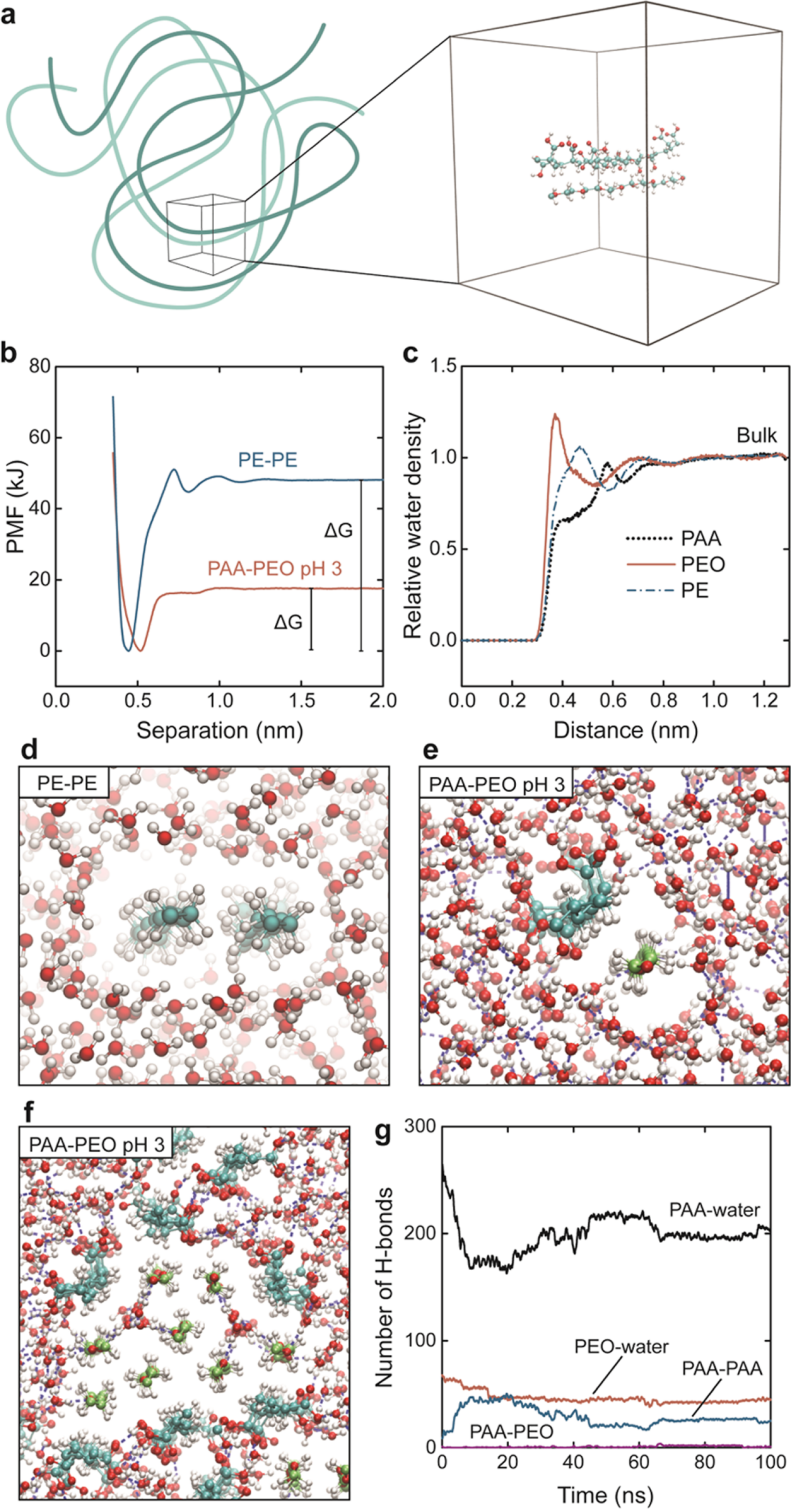
Molecular dynamics simulation data. (a) Schematic of how the computational model represents a short segment in a larger polymer complex. The segments were forced into an extended conformation by the boundary conditions, and their length was limited due to computational cost. (b) Potential of mean force (PMF) as a function of distance for a polyethylene pair and PAA–PEO at pH 3. (c) Relative water density as a function of (radial) distance from the polymer backbone. (d, e) Simulation snapshots from the simulations with marked hydrogen bonds for PAA–PEO. (f) Simulation of a larger complex of PEO and PAA. (g) Number of H-bonds associated with each polymer during the simulation in (f).

Sacrificing atomistic detail and using coarse-grained models was not an option here since important information about, e.g., hydrogen bonds would have been lost in the coarse-grained approximation. The most straightforward solution is to make the system smaller. Still, one problem is that short oligomers do not form complexes since their conformational and translational freedom is unrealistically large and they are too soluble (we tried this). Their conformational and translational freedom must be restricted for a short oligomeric segment to behave as a segment of a polymer coil (Figure 5a).

The conformational freedom of each monomer in a segment of a longer polymer chain is considerably restricted by the macroscopic structure of the coil. Thus, the polymers in our model were restricted to span between the walls of the simulation box. This setup permits the translation of the entire chain in three dimensions and rotation around its long axis while restricting internal degrees of freedom by enforcing an extended chain conformation (see the Experimental Section), which is undoubtedly too restricted. However, we chose a fully extended chain due to practical limitations in setting up a partially restricted chain segment. With this in mind, the reality is closer to a restricted chain than to a free oligomer, providing qualitative indications of the association mechanism. This was not an issue in previous simulations of entropy-driven adsorption to cellulose since the cellulose crystal is already in a highly restricted state that is easier to represent accurately at a smaller scale.^15^

We simulated the association of PAA and PEO at pH 3 and compared it to a purely hydrophobic self-association of polyethylene (PE). Figure 5b shows the potential of mean force (PMF) as a function of the separation between the polymer pairs, and Table S4 shows the thermodynamic parameters. Compared to the ITC results (Table 1), the simulated values for PAA–PEO have the correct sign and display negative free energy of association with a clear endothermic response of the same order of magnitude. However, they should not be expected to show quantitative agreement due to the limitations of the model setup and, to some extent, the empirical force-field parameters. Regardless, the entropy term is negative and thus drives the association. The question is whether a little more conformational freedom would change this, and we can not find a reasonable argument for this unless the complex is highly organized to have a perfect alignment of several favorable polar bonds, which has not been observed in the literature.

Due to the model setup, Δ*G* becomes relatively small, and since the entropy from the PMF simulation is calculated as Δ*G*-Δ*H*, the entropy is much smaller than that in experiments. To avoid this issue and calculate the entropy directly, we performed another simulation based on the two-phase thermodynamic method (2PT) to explicitly study the entropy of water.^33^ The entropy (−*T*Δ*S*) from this simulation was −20 kJ/mol for PAA–PEO or −30 kJ/mol for PE–PE (Table S4), which is in perfect agreement with experimental observations of around −20 to −30 kJ/mol (Tables S1 and S2). These results strongly support the idea that favorable entropy change comes from the reorganization of water.

PE–PE shows an oscillating potential of mean force as a function of separation, typical for hydrophobic effects,^30^ and solvophobic effects in general,^34^ when the structured hydration shells (Figure 4a) are formed/replaced. These oscillations are small or nonexistent for PAA–PEO at pH 3, and the free energy is about a third of that for PE–PE. Looking at the structuring of water with distance from the polymer (Figure 5c), PEO has tightly bound water close to the polymer, probably in H-bond configuration of ∼0.3 nm length. In contrast, for PE, there is a typical exclusion of water close to the polymer. PAA has a density spike at a 0.6 nm distance from the polymer backbone, which is the position of oxygen in the carboxylic acid groups plus the length of a H-bond. This water structuring agrees with the schematic in Figure 4a and shows that PEO and PAA interact favorably with water, at least in positions with oxygens, demonstrating their amphiphilic nature.

Looking at snapshots from the simulations, PE–PE forms a complex with the exclusion of the surrounding water (Figure 5d). The snapshot for PAA–PEO (Figure 5e) shows no interpolymer H-bonds (Table S5). Instead, all carboxylic groups face the water phase. Also, the PEO oxygens accept H-bonds from the water molecules. This configuration goes against many previous ideas of a hydrogen-bonded complex, as discussed above, and instead indicates that the PAA–PEO association could be closer to amphiphilic micelle formation.

Would more conformational freedom change this? We think not and instead argue that this snapshot is reasonable; why would strongly and favorably hydrated carboxylic acid groups face PEO in H-bonds and expose the less favorably hydrated backbone to water? Such behavior would go against the logic of the physical chemistry. Even if the chains had slightly higher conformational freedom, hiding polar groups in hydrogen bonds would still be very unfavorable: a complex morphology that simultaneously allows PAA–PEO hydrogen bonds, and the exposure of hydrophilic groups to water should not be possible. With more realistic conformational freedom, PEO would probably be even better hidden from contact with water.

A less hydrophilic polymer in PVME leads to a higher free energy gain by a greater entropy increase (Table 2), consistent with hiding less soluble polymer segments from water in a micelle-like structure. The picture is even more evident in a larger system with multiple chains (Figure 5f). The interpolymer hydrogen bonds that form are between PAA repeat units in the outer shell of the complex (Figure 5g).

The enthalpic change from PAA–H_2_O and PEO–H_2_O hydrogen bonds to PAA–PEO and H_2_O–H_2_O hydrogen bonds is, as mentioned, essentially zero. They may, however, form because none of the states are more favored, so they can exchange freely and dynamically if possible. Solid-state ^13^C NMR has been used to suggest three types of chemical environments for carboxylic acids inside extensively dried PAA–PEO complexes.^35^ Based on earlier infrared (IR) adsorption data, the authors suggested three types of hydrogen bonds:

(1) Complex form (PAA–PEO). 40–60% depending on the mixing ratio.
(2) Dimeric form (PAA–PAA). 30–40% depending on the mixing ratio.
(3) Free form (PAA-H_2_O). 5–20% depending on the mixing ratio.

PEO-H_2_O should also exist but could not be measured by using solid-state ^13^C NMR.

It is important to note that during extensive drying of the complexes, as for both IR and NMR measurements, water is driven out of the complex by heat, which favors PAA–PEO hydrogen bonds that may not exist in the wet complex at ambient conditions, due to a lack of other options. A dried complex is therefore a poor representation of the initial association mechanism, and this questionable reasoning is perhaps where the confusion about hydrogen-bond-driven association started. In a swollen state, although at a higher pH of 5, no complex hydrogen bonds were detected by ^13^C NMR even though the viscosity indicated weak complexation.^36^

Our simulations and logic of physical chemistry indicate that the initial association does not require hydrogen bonds. However, in their dimeric form, H-bonds can stabilize PAA facing the water phase. Carboxylic acids can later turn inward to form H-bonds with PEO, as suggested by IR and ^13^C NMR, increasing the entropy of water bound to oxygens of both polymers, i.e., dipole exchange. This scenario eventually occurs as the complex grows or is concentrated by dewatering. The water inside such a complex is in an unfavorable entropic state. If a PAA molecule is not in the outer shell of the complex facing water, it can form H-bonds, in their complex form, with PEO to release some of this water in a dipole exchange or when water is driven away by drying. Due to restrictions in the network, some water, in free form, will stay inside the complex, even after extensive drying, to form the hydrogen bonds that otherwise cannot form between the polymers. Removing this water would be extremely difficult. For PAA, this is suggested to be 5–20% of the carboxylic acids in the extensively dried state and is probably much more, if not most, in a wet complex.

Combining our simulation with IR and ^13^C NMR literature data, we suggest that although dipole exchange occurs, it is not required for PAA and PEO to associate at low pH in the first place. Complex-form H-bonds are just secondary relaxations through bond exchange. PAA–PEO could instead be considered to associate by amphiphilic association induced by the reduced solubility of PAAH compared to PAANa in combination with the hiding of PEO from water. The amphiphilic initial association in the simulation suggests that H-bond-driven polymer association in water is extremely rare if at all existing.

These results also raise the challenging question: what is the actual contribution of ion exchange in polyion association? Indeed, polyelectrolytes can associate at high salt concentrations where the gain of releasing counterions is essentially zero.^37^ For polyions, partial ion exchange is probably needed to minimize the repulsion in the complex. However, the charge exchange may enable a considerable entropic gain from amphiphilic association. For weak polyions with titrating groups, the amphiphilic association mechanism of PAA–PEO becomes more complex since protonation is another path to minimizing repulsion; this may be the reason for the biphasic salt-dependent behavior of PAA–PAH (Figure S1) and other weak polyion pairs. Recent coarse-grained MD simulations indicate that polyion association is driven by a reorganization of water rather than a release of counterions.^16^ More research is needed to understand the association of oppositely charged polymers.

### Recommendation for Future Use of Terminology for Polymer Research

Based on our findings, we make two concrete suggestions concerning the terminology used by scientists when describing or analyzing polymer association.

(i) Without clear thermodynamic signs for intermolecular bonds as the driving force, i.e., strongly exothermic interactions, refer to the process as “entropy-driven association.”
(ii) Do not use hydrophobic interactions or effects to describe the association of water-soluble polymers. Instead, call it “amphiphilic association,” highlighting that these complexes have a particular order.

## Conclusions and Outlook

Literature data, ITC measurements, and molecular dynamics simulations show that the association of polymer pairs in water is entropy-driven. To explain this, we propose a mechanism that includes charge exchange and amphiphilic association. This model unifies our description of the association of polymer in water. In particular, this dictates that specific intermolecular bonds, such as hydrogen bonds, are not required for polymer association. In endothermic cases, they even work against the association. Molecular dynamics simulations suggest that a charge exchange is unnecessary for the initial association and that amphiphilic association alone is enough. This mechanism should be further investigated for polyion pairs, where charge exchange is the accepted explanation. Polyion association may also originate from an amphiphilic association. However, during condensation of the complex, a charge exchange must occur.

This mechanism primarily applies to homopolymers or simple heteropolymers, which are dominant in many industrial chemical processes and in designing new sustainable materials. There are, however, cases of association in water driven by enthalpy, where the mechanism falls short. For example, small dyes or drugs whose entropy of adsorption is unfavorable as they have many degrees of freedom,^24^ or complex biological heteropolymers as in the cellulose-binding domains or protein–ligand interactions.^22,24,38^ Interestingly, many small organic molecules that associate due to a favorable enthalpy contain aromatic groups.^24,39,40^ The same can be observed for some oligomeric species, such as tannic acid, as shown in Table S2. As many as 99% of all drugs are composed of aromatic groups,^40^ by far the most characteristic chemical functionality. Also, aromatic groups such as catechols or quinones play an essential role in the underwater adhesives of marine organisms.^41^ The mechanisms behind the enthalpy-driven association of aromatic groups are unknown but probably related to their polarity and the distinct hydration pattern or their size, rendering their hydrophobic effect enthalpy-driven.^29,30^ To further develop a general model, studies on aromatic groups should be highly prioritized.

The mechanism presented here is an essential step toward understanding the association of polymers in water. These basic principles could also be extended to other solvents. Still, the energy landscape must be determined for each case since the enthalpy–entropy compensation is different depending on the properties of the solvent.

## Experimental Section

PEO (100 000 g/mol), PAA (100 000 g/mol), PVME (low, >12 000 g/mol), and PAH (65 000 g/mol) were purchased from Sigma-Aldrich, Sweden. The polymers were purified by dialysis using cellulose dialysis tubes with a molecular weight cutoff of 12 000 g/mol and subsequently freeze-dried. The dried samples were dissolved in Milli-Q water at 10 or 1 mM repeat-unit concentration, and the pH was adjusted using 0.1 or 0.01 M HCl and NaOH. The hydrodynamic size measured by DLS was 8–15 nm for these polymers in aqueous solution.

ITC measurements were conducted using an iTC200 Microcal instrument from GE Healthcare. In a typical measurement, the chamber was filled with a polymer solution with a repeat-unit concentration of 1 mM that was titrated by injecting 1–2 μL of a polymer solution with a repeat-unit concentration of 10 mM. At least three titrations were performed for each polymer pair. The injection duration was 3 s. The equilibrium time between injections was 120 s. The baseline temperature was 25 °C.

Thermograms were baseline adjusted, control subtracted, and fitted using the integrated public-domain software packages NITPIC, SEDPHAT, and GUSSI.^42^ For more information, we refer to the published protocol by Brautigam et al.^42^ The NITPIC software reads the raw data, baseline corrects it using a blank titration, and then integrates the peaks. The integrated data are then loaded into the SEDPHAT software. The first injection was discarded per standard, and the data was fitted using the software algorithm with built-in standard kinetic models. The algorithm requires an initial parameter guess and then fitted using a Marquardt–Levenberg algorithm until a minimum reduced chi-square was reached. We conducted several fittings for each sample with different starting parameters to reach a good fit with a low chi-square. Different kinetic models were tested. The integrated data and fitted model was loaded into GUSSI for plotting. Presented thermodynamic data are the averages of three separate titrations and fittings.

### MD Simulations

MD simulations were performed with GROMACS (version 2020.2 or later)^43^ using a Verlet integration algorithm with basic time step 2 fs. All nonbonded potentials were truncated at a distance of 1.2 nm and shifted to become zero at the cutoff. Long-range electrostatics were included using PME.^44^ Bonds in polymers were kept at their respective equilibrium values using P-LINCS,^45^ and water was kept completely rigid using SETTLE.^46^ Simulations were performed in the NVT ensemble with temperature maintained at 298 K using the velocity-rescale thermostat.^47^

The simulations employed models for PAA with protonated carboxylic acids (mimicking the conditions at pH 3) and PE of DP 12, and PEO of DP 8, which were generated using the Charmm-GUI^48^ using force-field parameters based on the Charmm General Force Field (CGenFF).^49^ The first and last monomer units were covalently linked, making them (effectively) infinitely long. Starting coordinates were generated in a straight all-trans configuration oriented parallel to the *z*-axis in a simulation box with dimensions of 4.5 × 4.5 × 3.0 nm^3^. The simulation box was subsequently filled with explicit Tip3p water molecules^50^ and energy-minimized. Due to the periodic boundary conditions, polymer chains are forced into an extended configuration and are thus slightly strained with respect to their respective equilibrium conformations. This strain will naturally affect the conformational space available for these polymer models and also, to some extent, the intermolecular interactions since it could exclude, for instance, specific H-bonding geometries. However, without pretending that these models are accurate descriptions of the real polymers, we argue that they serve as valid models for the local interactions between nonpolar chains (PE), chains that are H-bond acceptors (PEO), and fully H-bonding chains (PAA), in water.

Potential of mean force (PMF) calculations were performed using the accelerated weight histogram (AWH) method.^51^ The center-of-mass distance in the *x*/*y*-plane between two polymers was the reaction coordinate sampled between 0.4 and 2.0 nm. The force constant for the harmonic biasing potentials was 130 000 kJ mol^–1^ nm^–2^. The AWH simulations were run in triplets, each extending to 1 μs in length.

To calculate the enthalpic contribution to the PMF, additional equilibrium simulations of 100 ns were run for the aggregated system and two separate systems with the individual chains and half of the number of water molecules. The change in enthalpy was calculated as the difference in the total energy between the two cases.

The difference in entropy between the associated and dissociated states was calculated using the two-phase thermodynamics (2PT) method of the Goddard group,^33^ using our in-house implementation.^52^ The entropy estimate for each state was based on 10 independent 100 ps long simulations with velocities saved every 8 fs.

## Supporting Information Available

Abbreviations of polymer names; tables with literature, thermodynamic, DLS, and simulation data; weak polyion association; and extension of these principles to other solvents (PDF)
