# Supplementary material for: Entropy Drives Interpolymer Association in Water: Insights into Molecular Mechanisms
Source: Langmuir. 2024 Mar 22;40(13):6718–29. doi: 10.1021/acs.langmuir.3c02978 (PMC10993416; doi:10.1021/acs.langmuir.3c02978)
Supplement: Supplementary file 1 — la3c02978_si_001.pdf [file la3c02978_si_001.pdf]

Supplementary information

Entropy drives interpolymer association in water: insights into molecular mechanisms

Tobias Benselfelt, Goksu Cinar Ciftci, Lars Wågberg, Jakob Wohlert* and Mahiar Max Hamedi**

[1] Department of Fibre and Polymer Technology, School of Engineering Sciences in Chemistry, Biotechnology and Health, KTH Royal Institute of Technology, 100 44 Stockholm, Sweden

* Correspondence to: bense@kth.se, jacke@kth.se, mahiar@kth.se

Contents

List of polymer names 2

Table S1 3

Table S2 4

Figure S1 5

Weak polyions association – parallel mechanisms 6

Figure S2 7

Table S3 8

Polyion-polydipole interactions 9

Extending the charge exchange principles to other solvents 10

Figure S3 11

Table S4 12

Table S5 13

References 14

List of polymer names

PAA	Polyacrylic acid
PMAA	Polymethacrylic acid
PEO	Polyethylene oxide
PVME	Polyvinyl methyl ether
PAH	Polyallylamine hydrochloride
PDADMA	Polydiallyldimethylammonium (chloride)
PSS	Polystyrene sulfonate
SPEEK	Sulfonated polyether ether ketone
PGA	Polyglutamic acid
PLL	Poly-L-lysine
HA	Hyaluronic acid
PVS	Polyvinyl sulfate
CMP	Carboxymethyl pullulan
DEAE	Diethylaminoethyl dextran
PEI	Polyethyleneimine
PAsp	Polyaspartic acid
PO	Poly-L-ornithine hydrobromide
PHis	Polyhistidine
PVPo	Polyvinylpyrrolidone
MC	Methyl cellulose
HEC	Hydroxyethyl cellulose
TA	Tannic acid
PAM	Polyacrylamide

Table S1. Thermodynamics data of the association of polyion pairs and some smaller molecules at 25-30 °C and pH 5-8. c_{salt} is the salt concentration. Values are per mole of repeat units that can be charged. Exothermic associations, including at least one strong polyion, are marked red. Endothermic associations of weak polyion are marked blue. Pairs that do not match this division are marked white.

Polymer pair	c_{salt}	ΔH (kJ mol^{-1})	-TΔS (J mol^{-1} K^{-1})	ΔG (kJ mol^{-1})
PDADMA to PSS[1, 2]	0.1	–1.88	-	-
	0.3	–1.7	–1.0	–3
PSS to PDADMA[3]	0.15	–1.04	-	-
PAH to PSS[2-4]	0	–0.46	–32	–32
	0.01	–0.17	–30	–30
	0.3	–1.45	–10.0	–11
PSS to PAH[2-4]	0	–0.59	–31	–31
	0.01	–1.0	–33	–37
	0.15	–1.52	-	-
	2	–0.11	-	-
PDADMA to SPEEK[2]	0.3	+2.2	–10	–7.8
PGA to PAH[3]	0.16	+1.85	-	-
PGA to PAH[3]	0.001	–6.07	-	-
PGA to PLL[3]	0.15	+2.92	-	-
HA to PAH[3]	0.15	+0.64	-	-
HA to PLL[3]	0.15	+0.54	-	-
HA to PDADMA[3]	0.15	+1.60	-	-
PAA to PDADMA[5]	0	–1.67	-	-
PDADMA to PVS[6]	0.05	–2.2	–46	–49
CMP to DEAE dextran[7]	0.05[a]	+1.09 to 1.5	–30 to –33	–29 to –31
DEAE dextran to CMP[7]	0.05[a]	+0.69 to 0.95	–24 to –27	–23 to –26
P(glycine-co-lysine) to PGA[8]	0.025	~ +1.5	~ –27	~ –25
Citrate to tris-guanidinium 1:1[9]	0.05[b]	–2.0	–6.4	–6.4
Tris-guanidinium to citrate 1:1[9]	0.05[b]	–0.9	–6.3	–6.3
	0.103[b]	–0.3	–5.0	–5.0
PGA to PEI[10]	0.2	+2.4	–30	-28
PAsp to PEI[10]	0.2	+1.7	–30	-28
PGA to PO[10]	0.2	+3.0	–25	-22
PAsp to PLL[10]	0.2	+1.9	–29	-27
PAA to PAH[10]	0.2	+2.6	–32	-29
PGA to PHis[10]	0.2	-4.3	–19	-24
PGA to PO[10]	0	+8.9	–32	–23
	0.1	+3.7	–27	–23
	0.2	+3.0	–25	–22
	0.375	+2.4	–26	–23
	0.5	+2.2	–22	–20
	0.8	+0.9	–21	–20

c_s is NaCl unless noted by letters: [a] sodium acetate, [b] phosphate buffer

Table S2. Thermodynamics data of the association of polydipole pairs and some smaller molecules at 25-30 °C. Values are per mole of repeat units. Exothermic associations are marked red.

Polymer pair	pH	ΔH (kJ mol^{-1})	-TΔS (J mol^{-1} K^{-1})	ΔG (kJ mol$^{-1)}$
PMAA-PVPo[11]	3	5.9	-	-
PMAA-PEO[11]	3	1.3	-	-
PAA-PEO[11]	3	0.54	-	-
PAA - MC[12]	2	0.69	–19[b]	–18[a]
	2.3	0.58	–16[b]	–16[a]
	2.7	0.26	–6[b]	–6[a]
PMAA to HEC[13]	2	0.17	–16[b]	–16[a]
TA to HEC[13]	2	–3.25	–19[b]	–22[a]
PAA to PVA[13]	2	0.03	–12[b]	–12[a]
PMAA to PVA[13]	2	0.15	–22[b]	–22[a]
TA to PVA[13]	2	2.21	–24[b]	–22[a]
PAA to MC[13]	2	0.64	–20[b]	–19[a]
PMAA to MC[13]	2	0.51	–19[b]	–19[a]
TA to MC[13]	2	2.25	–25[b]	–23[a]
PAA to PEO[13]	2	0.57	–21 [b]	–20[a]
PMAA to PEO[13]	2	0.67	–21[b]	–21[a]
TA to PEO[13]	2	2.95	–21 [b]	–18[a]
PAA to PVPo[13]	2	0.31	–21[b]	–20 [a]
PMAA to PVPo[13]	2	0.35	–20[b]	–20 [a]
TA to PVPo[13]	2	–0.67	–18[b]	–19 [a]
PAA to PAM[13]	2	–1.20	–21[b]	–22 [a]
PMAA to PAM[13]	2	–1.06	–19[b]	–20 [a]
TA to PAM[13]	2	–8.25	–17[b]	–25 [a]
PAA to PVME[13]	2	1.54	–25[b]	–23 [a]
PMAA to PVME[13]	2	1.92	–25[b]	–23 [a]
TA to PVME[13]	2	12.44	–43[b]	–30 [a]

[a] estimated by ΔG = –RTlnk, [b] calculated by ΔG = ΔH –TΔS

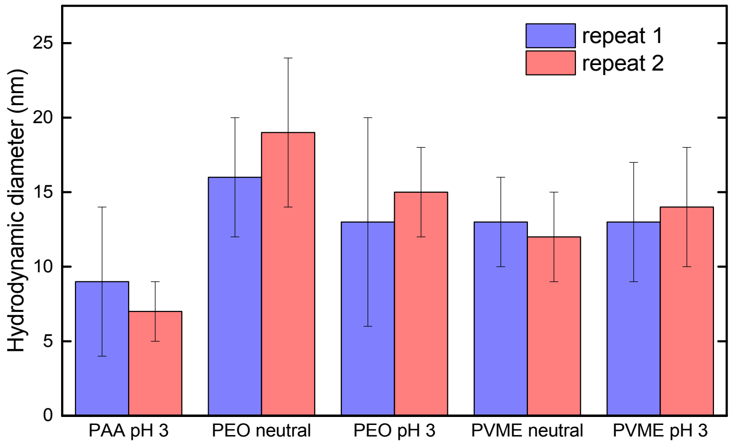

Figure S1. The hydrodynamic diameters of polymers in solution with a repeat unit concentration of 10 mM, measured using a Zetasizer ZEN3600, Malvern Instruments Ltd., U.K. The error bars are the standard deviation given by the software.

Weak polyions association – parallel mechanisms

We used ITC to study the association of one of the structurally simplest weak polyion pairs in PAA and polyallylamine hydrochloride (PAH) to complement the thermodynamic data of weak polyion association. Figure S2a-b shows that a background salt concentration of 100 or 10 mM NaCl considerably impacts the heat of association. In 10 mM (Figure S2a) the data suggest a biphasic association with an initial exothermic response which transitions into an endothermic response at about a 1:1 molar ratio of repeat units. In 100 mM (Figure S2b), the appearance is similar with the difference that the initial exotherm has lower heat release.

Biphasic behaviors have been observed before in the case of ITC measurements of the addition of polyvinyl amine (PVAm) to carboxymethyl cellulose (CMC) at 5 mM NaCl[6] or PEO-block-polylysine (PLL) to plasmid DNA at 10-600 mM NaCl.[14] The former pair, which behaved similarly to the data in Figure S2a, was not fitted with an interaction model since the opposite titration (CMC to PVAm) showed quite different behavior assigned to the nonequilibrium state of the association. The latter pair, which is more similar to the data in Figure S2b, was fitted using two endothermic binding events, one before and one after condensation of the DNA.[15]

In the case of PAA to PAH, the influence of salt indicates that the first binding should be explained by exothermic ion exchange since it saturates at about a 1:1 molar ratio and is sensitive to salt. The second binding is an endothermic dipole exchange or hydrophobic effect enabled by the available H-bond donor and acceptor sites or less hydrophilic segment on the formed neutral polyion complex.

Table S3 contains the thermodynamic parameters from the biphasic model for 10 mM NaCl. The 100 mM case was difficult to fit due to the sharp transition, and we avoided presenting values but note that the endotherm was very similar to that in 10 mM NaCl for most fits.

At higher salt concentrations, polyion association is less exothermic (Figure 2a) due to the screening of charged groups[1], and the data in Figures S2 and Table S3 agree with this trend. The second binding should not be affected by the salt concentration and is indeed similar to polydipole association (Figure 2b).

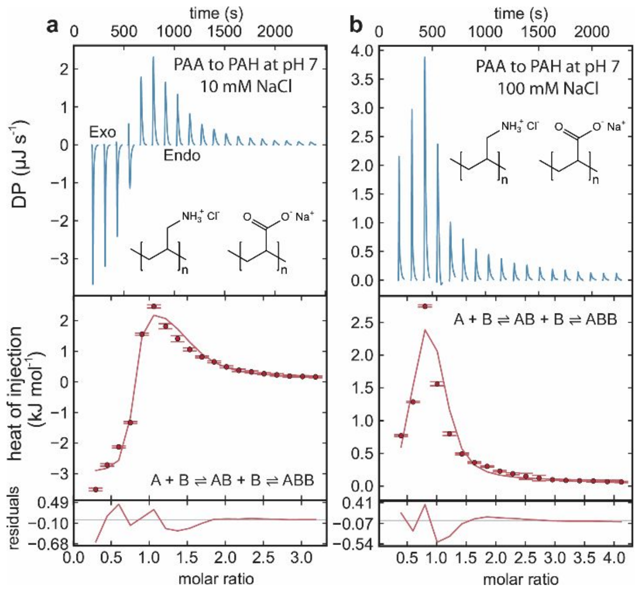

Figure S2. ITC data of the injection of 1 μL PAA 10 mM repeat units to 203 μL PAH 0.5 mM repeat units with a background salt concentration of: c) 10 mM or d) 100 mM NaCl both at pH 7 and 25 °C. Data point errorbars are errors from the integration and the solid line is the fitted model with the residuals for each data point shown below.

Table S3. Thermodynamic parameters for the association of PAA and PAH at pH 7 and 25 °C in 10 mM NaCl. The mean and standard deviation is from three separate fitted titrations.

		Exotherm			Endotherm		
Polymer pair		ΔH (kJ mol^{-1})	–TΔS (kJ mol^{-1})	ΔG (kJ mol^{-1})	ΔH (kJ mol^{-1})	–TΔS (kJ mol^{-1})	ΔG (kJ mol^{-1})
PAA to PAH	Mean (n=3)	–3.2	–34	–37	+3.5	–28	-25
	Std Dev	0.2	0.8	0.6	0.6	0.8	1.2

Polyion-polydipole interactions

The middle ground between ion and dipole exchanges is an ion-dipole exchange. This interaction is similar to the mechanism of dissolution of salts, i.e., the exchange of ionic bonds for ion-dipole bonds to increase the entropy of the salt ions, given that the solvent molecules are decent substitutes for the ionic bond. The more polar the solvent, the better. In this case, the ion can be a polyion, and the substitute can be a polar group of a polydipole. Although a complete ion exchange cannot be fully achieved, a dipole exchange can increase the entropy. If the counterion has a high charge density and is favorably dissociated and hydrated, it can be argued that the ion exchange is partially taking place (polyion-counterion + polydipole-water ⇌ polyion-polydipole + counterion-water).

PAA-PEO exists as "soluble" colloidal complexes above a critical pH of around 4 and as individual polymers above pH 12.[16] This behavior indicates that ion-dipole interactions are not significant for this polymer pair. It also shows that above pH 4, the repulsion between charged groups of PAA prevents further phase separation of the PAA-PEO complex, which is weakly associated by amphiphilic interactions.

Extending the charge exchange principles to other solvents

Two main criteria should be met for charge exchange: i) the solvent must have a strong polar interaction with the ions or dipoles of the polymers so that the interaction is not solvophobic. ii) the counterion or counterdipole must be able to form interactions with solvent molecules when released to fulfill the exchange, which generally means that the solvent is both a donor and acceptor of H-bonds (protic solvent).

The nature of the solvent, protic or aprotic, and the polarity have been shown to influence the complex formation of polymethacrylic acid (PMAA) and polyvinylpyrrolidone (PVPo) (Figure 6).[17] For protic solvents such as water or alcohols, the association decreases with decreasing the dielectric constant of the solvent. A lower polarity of the solvent results in weaker interaction and less confinement of solvent molecules, so the soluble state is not as entropically unfavored as in water. In contrast, the trend is the opposite for aprotic solvents, suggesting that the more polar the solvent, the higher the energy penalty of releasing the solvent molecules since they cannot form H-bonds in the released state. The solvent wins in this competition for H-bonds, probably due to its freedom to find the most optimal configuration.

The competition for hydrogen bonds has been shown by the association of PMAA and PVPo in different water/DMSO mixtures. This association was endothermic in pure water and transitioned to exothermic at around 25 vol% DMSO due to the favorable formation of DMSO-water complexes upon the release of DMSO.[18] In contrast to what the authors stated, the enthalpy of association was within $\pm$ 2.5 kJ mol^{-1} ($\pm$ k_BT) and could not drive the association.

PMAA formed H-bonds with DMSO at higher DMSO concentrations, and DMSO was not favorably exchanged to PVP, probably due to the diminishing opportunities to form new H-bonds in the released state as the water concentration is reduced (Figure 6). Indeed, the interaction was most exothermic at 50% DMSO, after which it turned back towards an endothermic situation at more than 70% DMSO when favorably bound DMSO had to be displaced.

Molecules or ions entering polymer complexes to break interactions are often called dopants. Some counterions or counterdipoles are better dopants, i.e., less favorable to release, preventing ion or dipole exchange.[1, 19] Each solvent/dopant system is unique, and discussing other solvents without detailed investigations is difficult. However, the dipole exchange mechanism is general and should contribute at least partially in other solvents than water.

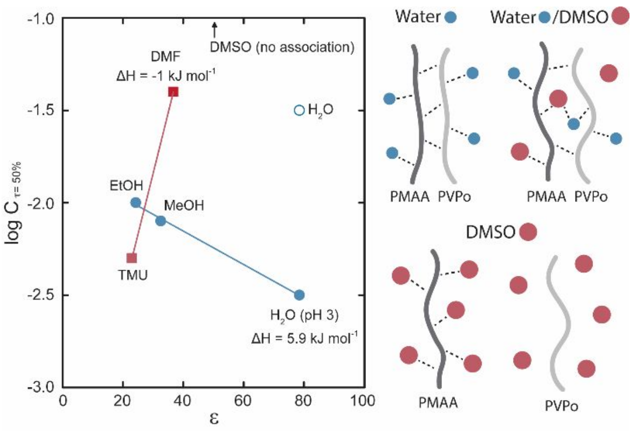

Figure S3. Data from ref [17] shows the degree of association (log $C_{\tau\,=\,50\%}$, which is the concentration at 50% transmittance) of PMAA and PVPo as a function of the dielectric constant of the solvent. The association is determined as the concentration where the transmittance reached 50%. TMU is tetramethylurea. The schematic is inspired by ref [18].

Table S4. Simulated thermodynamics parameters of the association of PAA and PEO at pH 3 and 25 °C.

	kJ mol^{-1} of segments			
System	ΔG (PMF)	ΔH (MD)	-TΔS (=ΔG-ΔH)	-TΔS_{water} (2PT)
PE-PE	-48.0 (0.4)	22.6 (2.9)	-70.6	-362 (53)
PAA-PEO	-17.6 (1.8)	40.3 (5.1)	-57.9	-162 (72)

	kJ mol^{-1} of repeat units			
System	ΔG (PMF)	ΔH (MD)	-TΔS (=ΔG-ΔH)	-TΔS_{water} (2PT)
PE-PE	-4	1.9	-5.9	-30
PAA-PEO	-2.2	5	-7.2	-20.3

Table S5. The number of H-bonds counted from the simulations.

System	H-bond type	No. H-bonds
PE	Water-water	1076
	Total	1076
PE-PE	Water-water	2151
	Total	2151
	Diff. (PE/PE-2*PE)	-1
PAA	Water-water	1029
	PAA-PAA	0
	PAA-water	29
	Total	1058
PEO	Water-water	1074
	PEO-water	8
	Total	1082
PAA-PEO	Water-water	2103
	PAA-PEO	0
	PAA-water	28
	PEO-water	7
	Total	2138
	Diff. ((PAA-PEO) - PAA - PEO)	-2

References

(1) Fu, J.; Schlenoff, J. B. Driving forces for oppositely charged polyion association in aqueous solutions: enthalpic, entropic, but not electrostatic. *Journal of the American Chemical Society* **2016**, *138* (3), 980-990.

(2) Bucur, C. B.; Sui, Z.; Schlenoff, J. B. Ideal Mixing in Polyelectrolyte Complexes and Multilayers: Entropy Driven Assembly. *Journal of the American Chemical Society* **2006**, *128* (42), 13690-13691.

(3) Laugel, N.; Betscha, C.; Winterhalter, M.; Voegel, J.-C.; Schaaf, P.; Ball, V. Relationship between the Growth Regime of Polyelectrolyte Multilayers and the Polyanion/Polycation Complexation Enthalpy. *The Journal of Physical Chemistry B* **2006**, *110* (39), 19443-19449.

(4) Bharadwaj, S.; Montazeri, R.; Haynie, D. T. Direct determination of the thermodynamics of polyelectrolyte complexation and implications thereof for electrostatic layer-by-layer assembly of multilayer films. *Langmuir* **2006**, *22* (14), 6093-6101.

(5) Alonso, T.; Irigoyen, J.; Iturri, J.; Moya, S. Study of the multilayer assembly and complex formation of poly (diallyldimethylammonium chloride)(PDADMAC) and poly (acrylic acid)(PAA) as a function of pH. *Soft Matter* **2013**, *9* (6), 1920-1928.

(6) Feng, X.; Leduc, M.; Pelton, R. Polyelectrolyte complex characterization with isothermal titration calorimetry and colloid titration. *Colloids Surf. Physicochem. Eng. Aspects* **2008**, *317* (1), 535-542.

(7) Le Cerf, D.; Pepin, A. S.; Niang, P. M.; Cristea, M.; Karakasyan-Dia, C.; Picton, L. Formation of polyelectrolyte complexes with diethylaminoethyl dextran: Charge ratio and molar mass effect. *Carbohydr. Polym.* **2014**, *113*, 217-224.

(8) Chang, L.-W.; Lytle, T. K.; Radhakrishna, M.; Madinya, J. J.; Vélez, J.; Sing, C. E.; Perry, S. L. Sequence and entropy-based control of complex coacervates. *Nature Communications* **2017**, *8* (1), 1273.

(9) Rekharsky, M.; Inoue, Y.; Tobey, S.; Metzger, A.; Anslyn, E. Ion-pairing molecular recognition in water: Aggregation at low concentrations that is entropy-driven. *Journal of the American Chemical Society* **2002**, *124* (50), 14959-14967.

(10) Priftis, D.; Laugel, N.; Tirrell, M. Thermodynamic Characterization of Polypeptide Complex Coacervation. *Langmuir* **2012**, *28* (45), 15947-15957.

(11) Tsuchida, E.; Abe, K. Interactions between macromolecules in solution and intermacromolecular complexes. In *Interactions between macromolecules in solution and intermacromolecular complexes*, Springer, 1982; pp 1-119.

(12) Khutoryanskaya, O. V.; Williams, A. C.; Khutoryanskiy, V. V. pH-Mediated Interactions between Poly(acrylic acid) and Methylcellulose in the Formation of Ultrathin Multilayered Hydrogels and Spherical Nanoparticles. *Macromolecules* **2007**, *40* (21), 7707-7713.

(13) Bizley, S. C.; Williams, A. C.; Khutoryanskiy, V. V. Thermodynamic and kinetic properties of interpolymer complexes assessed by isothermal titration calorimetry and surface plasmon resonance. *Soft Matter* **2014**, *10* (41), 8254-8260, 10.1039/C4SM01138D.

(14) Kim, W.; Yamasaki, Y.; Jang, W.-D.; Kataoka, K. Thermodynamics of DNA Condensation Induced by Poly(ethylene glycol)-block-polylysine through Polyion Complex Micelle Formation. *Biomacromolecules* **2010**, *11* (5), 1180-1186.

(15) Kim, W.; Yamasaki, Y.; Kataoka, K. Development of a Fitting Model Suitable for the Isothermal Titration Calorimetric Curve of DNA with Cationic Ligands. *The Journal of Physical Chemistry B* **2006**, *110* (22), 10919-10925.

(16) Bailey Jr., F. E.; Lundberg, R. D.; Callard, R. W. Some factors affecting the molecular association of poly(ethylene oxide) and poly(acrylic acid) in aqueous solution. *Journal of Polymer Science Part A: General Papers* **1964**, *2* (2), 845-851.

(17) Ohno, H.; Abe, K.; Tsuchida, E. Solvent effect on the formation of poly(methacrylic acid)-poly(N-vinyl-2-pyrrolidone) complex through hydrogen bonding. *Die Makromolekulare Chemie* **1978**, *179* (3), 755-763.

(18) Selin, V.; Aliakseyeu, A.; Ankner, J. F.; Sukhishvili, S. A. Effect of a Competitive Solvent on Binding Enthalpy and Chain Intermixing in Hydrogen-Bonded Layer-by-Layer Films. *Macromolecules* **2019**, *52* (12), 4432-4440.
(19) Ghostine, R. A.; Shamoun, R. F.; Schlenoff, J. B. Doping and Diffusion in an Extruded Saloplastic Polyelectrolyte Complex. *Macromolecules* **2013**, *46* (10), 4089-4094.
